# Assessment and Application of the Hear Score in Remote Emergency Medicine Outposts in Bosnia and Herzegovina

**DOI:** 10.3390/medicina60040657

**Published:** 2024-04-19

**Authors:** Armin Šljivo, Nemanja Lukić, Aladin Altic, Slobodan Tomić, Arian Abdulkhaliq, Leopold Reiter, Diana Maria Bota, Eljakim Mahendran, Wisam Natour, Fatima Gavrankapetanović, Emira Kapisazović, Haris Duljević, Lana Lekić, Dragana Radoičić, Sanja D Tomić

**Affiliations:** 1 Clinical Center of University of Sarajevo, 71000 Sarajevo, Bosnia and Herzegovina; 2 University Clinical Center of the Republic of Srpska, 78000 Banja Luka, Bosnia and Herzegovina; 3 Dom Zdravlja Bihac, 77000 Bihac, Bosnia and Herzegovina; aladin.altic@gmail.com; 4 Faculty of Medicine, University of Novi Sad, 21000 Novi Sad, Serbiasanja.tomic@mf.uns.ac.rs (S.D.T.); 5 Faculty of Medicine, Iuliu Haţieganu University of Medicine and Pharmacy Cluj-Napoca, 4000348 Cluj-Napoca, Romania; 6 General Hospital Abdulah Nakaš, 71000 Sarajevo, Bosnia and Herzegovina; 7 Faculty of Health Studies, University of Sarajevo, 71000 Sarajevo, Bosnia and Herzegovina; 8 Institute for Cardiovascular Disease Dedinje, 11000 Belgrade, Serbia

**Keywords:** angina pectoris, myocardial infarction, HEAR score, emergency department, risk score

## Abstract

*Background and Objectives*. In emergency departments, chest pain is a common concern, highlighting the critical importance of distinguishing between acute coronary syndrome and other potential causes. Our research aimed to introduce and implement the HEAR score, specifically, in remote emergency outposts in Bosnia and Herzegovina. *Materials and Methods.* This follow-up study conducted a retrospective analysis of a prospective cohort consisting of patients who were admitted to the remote emergency medicine outposts in Canton Sarajevo and Zenica from 1 November to 31 December 2023. *Results*. This study comprised 103 (12.9%) patients with low-risk HEAR scores and 338 (83.8%) with high-risk HEAR scores, primarily female (221, 56.9%), with a mean age of 63.5 ± 11.2). Patients with low-risk HEAR scores were significantly younger (50.5 ± 15.6 vs. 65.9 ± 12.1), had fewer smokers (*p* < 0.05), and exhibited a lower incidence of cardiovascular risk factors compared to those with high-risk HEAR scores. Low-risk HEAR score for prediction of AMI had a sensitivity of 97.1% (95% CI 89.9–99.6%); specificity of 27.3% (95% CI 22.8–32.1%); PPV of 19.82% (95% CI 18.67–21.03%), and NPV of 98.08% (95% CI 92.80–99.51%). Within 30 days of the admission to the emergency department outpost, out of all 441 patients, 100 (22.7%) were diagnosed with MACE, with AMI 69 (15.6%), 3 deaths (0.7%), 6 (1.4%) had a CABG, and 22 (4.9%) underwent PCI. A low-risk HEAR score had a sensitivity of 97.0% (95% CI 91.7–99.4%) and specificity of 27.3% (95% CI 22.8–32.1%); PPV of 25.5% (95% CI 25.59–28.37%); NPV of 97.14% (95% CI 91.68–99.06%) for 30-day MACE. *Conclusions*. In conclusion, the outcomes of this study align with existing research, underscoring the effectiveness of the HEAR score in risk stratification for patients with chest pain. In practical terms, the implementation of the HEAR score in clinical decision-making processes holds significant promise.

## 1. Introduction

Chest pain is a common health issue frequently encountered by healthcare professionals in diverse medical fields which can be linked to a range of cardiac and non-cardiac conditions. Usually, it is described as a sensation of pressure or heaviness in the chest, or as discomfort that may vary in both intensity and duration [1]. In certain studies, conducted in the United States, it was found that nearly 80% of individuals seeking medical attention for this health concern receive a diagnosis that does not involve acute coronary syndrome (ACS), while the remaining 20% fall within a spectrum that includes conditions like unstable angina pectoris to acute myocardial infarction (AMI) [2,3]. Annually, around 2% to 5% of individuals with ACS are mistakenly discharged from emergency departments, and the failure to identify ACS cases represents a significant portion of medical malpractice claims [4].

Although various anamnestic information, electrocardiogram (ECG), and initial laboratory findings are valuable in identifying patients with ACS, the reliability becomes limited in patients who have concurrent comorbidities such as chronic kidney disease, systemic inflammation, viral myocarditis, infiltrative disease such as sarcoidosis, previous surgical and interventional cardiac procedures, and even acute stroke. Furthermore, older patients and those with diabetes mellitus due to impaired neurological responses exhibit different symptoms in ACS, mimicking other diagnoses and hindering timely diagnosis and treatment. In such cases, the utilization of a risk score becomes essential, as it can serve as a valuable tool to predict the likelihood of developing ACS in the future [5]. 

Currently these include the Thrombolysis in Myocardial Infarction score (TIMI), the Emergency Department Assessment of Chest Pain score (EDACS), the History, ECG, Age, Risk factors and Troponin (HEART) score and its derivate History, ECG, Age and Risk factors (HEAR) score. The TIMI score assesses the 14-day risk of major adverse cardiac events (MACE), and while it is supported by the American Heart Association and the American College of Cardiology, certain studies present conflicting findings, particularly regarding its standalone application [6]. Some researchers even propose that it cannot be used as a proper screening tool for ruling out low-risk patients in emergency departments [7]. The EDACS score, on the other hand, not only shows effective discriminatory capacity for ACS but also, when integrated into routine triage, has the potential to improve performance by identifying nearly half of the patients with potential cardiac chest pain as having a low risk of short-term major adverse cardiac events [8,9]. Finally, the HEART score [10,11] with its superior discriminative ability in distinguishing between patients with and without MACE places the particular focus on short-term outcomes. It successfully identified a substantial group of low-risk patients while maintaining a comparable level of safety in the stratification of risk [12]. A HEART score falling within the range of 0 to 3 designates a patient as “low risk” for MACE and may be considered for discharge; scores between 4 and 6 are categorized as “moderate risk”, and for them, admission for further testing is advised, while scores of 7 to 10 are classified as “high risk” and should undergo evaluation for potential invasive testing. In the setting of primary health care and remote emergency medicine outposts where troponin is not available, the derivate from the HEART score, the History, ECG, Age and Risk factors (HEAR) score, could be utilized [13]. The HEAR score effectively detected patients at a low risk of experiencing MACE and it could aid healthcare professionals in determining which patients would benefit most from troponin testing. According to Stopyra et al., the low modified HEART score (without troponin) has a high negative predictive value for MACE within 30 days, which implies a strong ability of this scoring system to rule out the occurrence of MACE within the specified timeframe [14]. Similarly, Khaleghi Rad et al., in a recent meta-analysis, also found the same results, with a low HEAR score exhibiting high negative predictive value reaching as high as 99.8% for MACE [13]. Moreover, the HEAR score could help in determining the most suitable location for care, whether it be a Cath-lab capable center, the nearest emergency department, or a cardiology clinic. Even though some researchers such as Dongen et al. [15] reported that prehospital measurement of troponin increased the predictive value of the HEART score for MACE compared to the HEAR score, and Stopyra et al. [16] indicated that in some settings the HEAR score may not always be adequate for guiding prehospital decision making in patients with chest pain, in settings of limited medical resources, it may provide guidance on the urgency for further diagnostics, treatment options, and transportation options, including options like activating emergency medical services, self-transport, or making a semi-urgent referral to cardiology clinics.

Our research aimed to introduce and implement the HEAR score, specifically, in remote primary care outposts in Bosnia and Herzegovina, after the presentation of the HEART score to the emergency medical departments of Bosnia and Herzegovina [17]. The subsequent phase involves gathering data on the prevalence of chest pain conditions, morbidity and mortality related to heart disease, and the outcomes based on the HEAR score. By introducing the HEAR score, out study aims to minimize everyday malpractice, to achieve timely diagnosis and treatment of ACS and to standardize the cardiac chest pain diagnosis in Bosnia and Herzegovina.

## 2. Materials and Methods

This follow-up study conducted a retrospective analysis of a prospective cohort consisting of patients who were admitted to the remote emergency medicine outposts in Canton Sarajevo and Zenica during the period from 1 November to 31 December 2023. To guarantee compliance with ethical guidelines and proper research procedures, this study obtained approval from the Bioethical Committee of Dom Zdravlja Zenica (OU-01.1-99-40435/22, 26 July 2022). This investigation was carried out in alignment with the pertinent amendments of the Helsinki Declaration, which delineates ethical principles for medical research involving human participants. This commitment aimed at safeguarding the rights, privacy, and confidentiality of patients throughout the entire study.

### 2.1. Subjects

The individuals enrolled in this study were patients who came to remote emergency medicine outposts’ departments in Sarajevo and Zenica due to chest pain. The minimum sample size was determined using the Cochran formula for the populations of Canton Sarajevo and the urban area of Zenica, resulting in a requirement of 384 patients with a margin of error set at 5%. Acceptance/Inclusion criteria encompassed (i) patients presenting with chest pain at remote emergency medicine outposts in Sarajevo and Zenica; (ii) willingness to participate in this study and provide informed consent after receiving comprehensive information about the study’s objectives and its voluntary nature; (iii) absence of a diagnosis of ST elevation myocardial infarction at the time of enrollment; (iv) patients who remain stable and do not experience fatal events within the emergency medical outposts during the time of enrollment. Rejection/Exclusion criteria encompassed (i) patients with ST elevation myocardial infarction; (ii) those leaving clinics against medical advice; (iii) individuals declining or not being able to provide research consent; (iv) those transferred to other regional hospitals through emergency medical outposts’ cardio mobiles. Before participating, all subjects received thorough information about the study’s objectives, understanding its voluntary nature, and were required to give informed consent. Additionally, patients were provided with detailed information about the specific data collected for study purposes.

### 2.2. Study Instruments and Data Collection

Clinical data were systematically gathered using a standardized case report form compliant with established clinical data standards. Skilled healthcare practitioners, including experienced clinicians, residents, and medical doctors with clinical experience, compiled comprehensive information for the HEAR score. This encompassed patient history, electrocardiogram (ECG) findings, age, and risk factors.

Post-assessment, patient dispositions were categorized as either discharge or undischarged, the latter involving hospitalization or referral to a cardiologist. Throughout the 30-day follow-up, data were meticulously collected from both digital and written patient records, including discharge letters, revascularization reports, and other pertinent medical documentation. When follow-up information was unavailable in hospital records, proactive measures were taken, including direct communication with the patient or their general practitioner. This was carried out to establish contact and gather comprehensive details about the patient’s medical condition, hospital admissions, occurrences of myocardial infarction, and revascularization procedures. Major Adverse Cardiovascular Events (MACE) were defined as a composite outcome, encompassing cardiovascular death, acute myocardial infarction (AMI), and revascularization, whether through percutaneous coronary intervention (PCI) or coronary artery bypass grafting (CABG) [17]. The selection of a 30-day follow-up period is crucial for capturing immediate post-acute events and complications that might occur shortly after an index event or intervention. This duration facilitates a comprehensive assessment of the direct impact of the treatment or intervention under scrutiny. Opting for a more extended follow-up could introduce biases from factors like loss to follow-up, changes in treatment modalities, or unrelated events, potentially confounding the study outcomes. A shorter follow-up period minimizes these confounding factors, ensuring a more focused and accurate collection of medical data.

The HEAR score aligns with the initial four components of the HEART score [10,11,13], each assigned a rank from 0 to 2, resulting in an overall score ranging from 0 to 8. A predefined threshold of two is established, distinguishing a low HEAR score (0 or 1) from a high HEAR score (2 to 8). The determination of a low HEAR score was initially based on its ability to effectively rule out major adverse cardiac events within 45 days [18]. 

The lack of specific details regarding chest pain, including its pattern, onset, duration, association with exercise, stress, or cold, location, accompanying symptoms, and response to sublingual nitrates, resulted in categorizing the medical history as “non-specific” with a score of zero points. A combination of non-specific and suspicious elements led to a classification of “moderately suspicious” with one point, while primarily specific elements indicated a “highly suspicious” status with two points.

Regarding the ECG categorization, an ECG meeting the Minnesota criteria for “normal” was assigned zero points. In cases with repolarization abnormalities lacking notable ST-segment depression, the presence of a bundle branch block, typical abnormalities suggesting left ventricular hypertrophy, repolarization abnormalities likely due to digoxin use, or unchanged known repolarization disturbances, one point was assigned. Two points were given for significant ST-segment depressions or elevations in the absence of a bundle branch block, left ventricular hypertrophy, or digoxin use.

The patient’s age upon admission was taken into account, with zero points assigned if the patient was younger than 45 years, one point for ages between 45 and 65 years, and two points for patients aged 65 years or older.

To evaluate the risk factors for coronary artery disease, the count included currently treated diabetes mellitus, current or recent smoking, diagnosed hypertension, diagnosed hypercholesterolemia, a family history of coronary artery disease, and obesity. If none of these risk factors were present, zero points were assigned; one or two risk factors resulted in one point, and three or more risk factors were awarded two points. Additionally, a history of coronary revascularization, myocardial infarction, stroke, or peripheral arterial disease resulted in two points.

After the initial assessment, we categorized the patients into two groups: those with a low-risk HEAR score and those with a high-risk HEAR score. Patients with a high-risk HEAR score were either recommended for observation or directed towards secondary or tertiary health care institutions for additional cardiological tests, both invasive and noninvasive, to discern the origin of the chest pain—determining whether it was associated with cardiac ischemia, non-ischemic cardiac problems, or if it mimicked other medical conditions.

### 2.3. Statistical Analysis

In the statistical analysis, collected data underwent examination and summarization through descriptive statistics. For data that followed a normal distribution, outcomes were expressed in frequencies and percentages, along with the mean ± standard deviation. Non-normally distributed data were presented as the median, accompanied by the 25th and 75th percentiles. Various statistical tests, such as independent samples *t*-tests, Mann–Whitney U tests, or chi-square tests, were employed to assess the relationship between different variables and specific phenomena. The statistical significance level for all tests was set at *p* < 0.05, utilizing a two-sided approach. 

## 3. Results

In this study, a total of 441 patients were included in the study setting, after excluding nine cases because of the exclusion criteria. Our population was predominately female, 221 (56.9%), with a mean age of 63.5 ± 11.2, with hypertension 347 (78.6%), diabetes mellitus 118 (26.7%), hyperlipidemia 303 (68.7%), BMI > 25 kg/m^2^ 125 (28.3%), and 145 (32.8%) were smokers. 

The sample included 103 (12.9%) patients with a low-risk HEAR score and 338 (83.8%) with a high-risk HEAR score. Patients with a low-risk HEAR score were significantly (*p* < 0.001) younger (50.5 ± 15.6 vs. 65.9 ± 12.1), had fewer smokers (*p* < 0.05), and had lower incidence of cardiovascular risk factors when compared to high-risk HEAR score patients. In contrast, patients with a high-risk HEAR score exhibited a predominance of male gender (X^2^ = 30.12, *p* < 0.001). All other demographic data (sex, age) and cardiovascular-associated risk factors are presented in Table 1. 

All patients who were admitted to remote emergency medicine outposts due to chest pain underwent a thorough examination of their medical history and details concerning the nature of the chest pain. Additionally, the analysis incorporated the review of their previous laboratory results from visits to other medical institutions.

On the previous laboratory report, high-risk HEAR score patients had a significantly (*p* < 0.05) higher mean score of creatinine (120.5 ± 45.2 vs. 88.3 ± 27.6) when compared to low-risk HEAR score patients. There was no significant (*p* > 0.05) difference in the mean systolic and diastolic pressure between the groups of low-risk HEAR score and high-risk HEAR score patients. Patients presenting with ECG alterations, including nonspecific repolarization changes or ST depression, showed a statistically significant association (*X*^2^ = 102.33, *p* < 0.001) with the high-risk HEAR score group, and such patients were subsequently either closely observed or referred to secondary or tertiary healthcare institutions for further investigation. When laboratory results were compared between low-risk and high-risk HEAR score, there were no significant differences, except in term of creatinine results (*p* = 0.043). All other laboratory, blood pressure, and ECG findings among low-risk HEAR and high-risk HEAR score patients are presented in Table 2.

Out of the whole study sample, 338 (76.6%) high-risk HEAR patients were referred for further cardiovascular investigation (troponin, echocardiography, coronary angiography, etc.). In secondary or tertiary healthcare facilities, among patients with high-risk HEAR scores, 236 patients (69.8%) exhibited troponin levels within the normal range, 58 patients (17.1%) had levels one to three times higher than normal, and 44 patients (13.1%) showed troponin levels exceeding three times the normal range. What was observed in the low-risk HEAR patients within 30 days of the remote emergency outpost admission is that 20 (19.4%) patients were referred for troponin assessment and only 3 had troponin which was one to three times higher than normal. High-risk HEAR score patients had significantly (X^2^ = 32.34, *p* < 0.001) higher troponin levels when compared to low-risk HEAR score patients. 

Among high-risk HEAR score patients referred for further cardiovascular investigation, 67 (19.8%) had AMI, 24 (7.0%) unstable angina, 8 (2.3%) arrythmia, and 3 (0.9%) other cardiac diseases. Low-risk HEAR score for prediction of AMI had a sensitivity of 97.1% (95% CI 89.9–99.6%); specificity of 27.3% (95% CI 22.8–32.1%); PPV of 19.82% (95% CI 18.67–21.03%), and NPV of 98.08% (95% CI 92.80–99.51%). Within 30 days of the admission to the emergency department outpost, out of all 441 patients, 100 (22.7%) were diagnosed with MACE, with AMI 69 (15.6%), 3 deaths (0.7%), 6 (1.4%) had a CABG, and 22 (4.9%) underwent PCI. Among patients with a low HEAR score, 2.9% (3/103 with 2 AMI and 1 PCI) had MACE compared to patients with a high-risk HEAR score at 28.7% (97/338 with 67 AMI, 3 deaths, 5 had a CABG, and 21 underwent PCI). Patients with low-risk HEAR score had a significantly (X^2^ = 26.45, *p* < 0.001) lower rate of MACE when compared to high-risk HEAR score patients. A low-risk HEAR score had a sensitivity of 97.0% (95% CI 91.7–99.4%) and specificity of 27.3% (95% CI 22.8–32.1%); PPV of 25.5% (95% CI 25.59–28.37%); NPV of 97.14% (95% CI 91.68–99.06%) for MACE. Furthermore, low-risk HEAR score patients had less suspicious patient history (X^2^ = 116.67, *p* < 0.001) and less prevalent cardiovascular risk factors (X^2^ = 193.26, *p* < 0.001) than high-risk HEAR score patients. Three deaths observed in this study are of cardiac cause and in the high-risk HEAR group. All other information regarding certain entities of patient history, ECG findings, age, risk factors, and between low-risk HEAR and high-risk HEAR score patients are presented in Table 3.

## 4. Discussion

To the readers’ knowledge, this is a subsequent study regarding implementation of the HEART and HEAR score in emergency departments and its outposts in Bosnia and Herzegovina, as well as the 30-day adverse cardiac events of these tools in emergency departments’ outposts. Our study sample predominantly comprised older individuals of normal weight, with an equal representation of both genders who had risk factors such as hypertension, diabetes mellitus, hyperlipidemia, and who were smokers. Patients with a low-risk HEAR score were younger and had a lower incidence of cardiovascular risk factors when compared to high- risk HEAR score patients, while patients with a high-risk HEAR score were more male, had higher troponin levels when admitted to the hospital, had higher rates of moderately to highly suspicious chest pain history, and more ECG changes. Within 30 days of the admission to the emergency department outpost, a small proportion of patients were diagnosed with MACE, including AMI, death, had a CABG or underwent PCI. Patients with a low-risk HEAR score had a lower rate of MACE compared to a high-risk HEAR score. The low-risk HEAR score had high sensitivity and low specificity for the detection of AMI and 30-day MACE.

In terms of demographic data, the high-risk HEAR score was more prevalent among males, patients who smoke, and older patients, which is consistent with previous studies [12]. Additionally, high-risk patients had a higher incidence of cardiovascular risk factors, such as hypertension, diabetes mellitus, hyperlipidemia, history of stroke, peripheral vascular disease, smoking, and a positive family history of cardiovascular disease, compared to the low-risk group. These results emphasize the complex connection between conventional cardiovascular risk factors and an elevated HEAR score, validating the discriminative ability of the HEAR score in detecting the existence of these risk factors and understanding their influence on a comprehensive risk assessment.

The laboratory findings in this study contribute compelling evidence to reinforce the discriminative efficacy of the HEAR score. Among patients, those with high-risk HEAR scores exhibited significantly elevated levels of creatinine compared to their low-risk counterparts. Creatinine, a biomarker associated with renal impairment, is commonly linked to acute coronary syndrome and cardiovascular diseases [11,12], often attributed to inadequate kidney perfusion, and presents an independent predictor of hospital death and major bleeding [19]. Furthermore, the higher prevalence of high-risk HEAR scores among older patients underscores a noteworthy connection between renal impairment and advancing age, a correlation frequently observed in clinical contexts [20].

Patients manifesting ECG aberrations, notably nonspecific repolarization changes or ST depression, displayed a statistically significant association with the high-risk HEAR score group. This intricate relationship underscores the potential clinical implications of specific ECG alterations as markers for heightened cardiovascular risk in patients [21]. The observed correlation strengthens the diagnostic utility of the HEAR score, affirming its capacity to discern patients with distinct ECG patterns that may signify an increased susceptibility to adverse cardiac events. 

The results from secondary or tertiary healthcare facilities offer a valuable perspective on troponin levels among patients with high-risk HEAR scores, emphasizing positive implications for clinical practice [17]. It is noteworthy that a substantial proportion of these high-risk HEAR score patients exhibited troponin levels within the normal range. This finding underscores the potential of the high-risk HEAR score as a sensitive tool, preventing oversight in the diagnosis of ACS. Even in cases where troponin levels are not markedly elevated, the high-risk HEAR score serves as an effective indicator, prompting further assessment. This nuanced categorization is instrumental in critical triage decisions, particularly in settings with limited diagnostic resources. In scenarios where comprehensive assessments might be challenging, the high-risk HEAR score becomes a crucial tool for prioritizing patients with chest pain, ensuring that those at potential risk of coronary syndrome are promptly referred for troponin assessment and HEART score evaluation. This strategic approach enhances the efficiency of patient triage, facilitating timely intervention and appropriate allocation of resources for optimal patient care [22].

The low-risk HEAR score emerges as a valuable tool for clinicians, demonstrating commendable sensitivity in predicting AMI and 30-day MACE. This characteristic ensures that the majority of individuals genuinely experiencing AMI are identified, minimizing the risk of oversight and ensuring a robust diagnostic approach [18]. The sensitivity, in this context, is crucial as it aids clinicians in not missing the diagnosis of acute coronary syndrome, a pivotal step in timely and effective patient management. Additionally, the PPV underscores that among those identified as high risk by the HEAR score, a significant proportion truly experiences AMI. This implies that the high-risk HEAR score serves as a reliable indicator, allowing clinicians to confidently initiate appropriate interventions and allocate resources for individuals at an elevated risk of adverse cardiovascular outcomes. Moreover, the high specificity of the NPV reinforces the reliability of the HEAR score in excluding individuals without AMI. This feature ensures that clinicians can confidently rule out AMI in patients with low-risk HEAR scores, streamlining decision making and avoiding unnecessary interventions [13].

The observed incidence of MACE within 30 days post-admission further accentuates the clinical relevance of the low-risk HEAR score. The predictive power of this score not only aids in diagnosing acute coronary syndrome but also plays a preventive role by identifying individuals at risk of MACE [13]. Our findings align with previous research [13,14,23], affirming the HEAR score’s effectiveness in excluding the likelihood of MACE within a defined timeframe. This dual functionality enhances the clinical utility of the HEAR score, making it a valuable tool for clinicians in risk stratification and patient management. The low-risk HEAR score proves to be a beneficial ally for clinicians, ensuring a heightened sensitivity to diagnose acute coronary syndrome and preventing MACE in the critical 30-day post-admission window. Its reliability in identifying individuals at risk underscores its potential as an indispensable component of cardiovascular risk assessment in clinical practice [13].

While acknowledging the strengths of this study and the corroborative evidence from prior research, it is crucial to acknowledge certain limitations. This study was conducted within a specific setting, potentially limiting its representativeness to the diverse patient populations and healthcare systems found in other regions. Consequently, there is a need for additional research as some studies [15,16] present that sometimes the score is insufficient to guide prehospital decision making in patients with chest pain. 

This study significantly contributes to the current literature by providing comprehensive insights into the effectiveness of the HEAR score in predicting adverse cardiac events within a specific healthcare context. By examining a diverse patient population in Bosnia and Herzegovina’s emergency departments, this study validates the utility of the HEAR score in identifying individuals at heightened risk of acute cardiac events, such as AMI and unstable angina. Furthermore, this study’s findings regarding the sensitivity, specificity, PPV, and NPV of the HEAR score add valuable quantitative data to the existing literature, enhancing our understanding of its clinical applicability. Overall, this study contributes substantively to the literature by providing evidence-based guidance for clinicians and researchers involved in cardiovascular risk assessment and management.

## 5. Conclusions

In conclusion, the outcomes of this study align with existing research, underscoring the effectiveness of the HEAR score in risk stratification for patients with chest pain. The study data, incorporating the HEAR score, revealed that it effectively identified high-risk patients, as reflected in their troponin levels and subsequent MACE within the 30-day post-admission period. This aligns with the broader utility of the HEAR score in cardiovascular risk assessment. In practical terms, the implementation of the HEAR score in clinical decision-making processes holds significant promise. It not only aids in distinguishing between patients at varying levels of risk but also facilitates appropriate triage and management strategies. The data-driven insights from the HEAR score provide clinicians with a valuable tool to enhance patient care and optimize resource allocation, emphasizing its potential as a valuable component in cardiovascular risk assessment protocols.

## Figures and Tables

**Table 1:** Sex, age, and cardiovascular-associated risk factors such as hypertension, diabetes mellitus, hyperlipidemia, smoking status, and positive family history of cardiovascular disease among low-risk HEAR and high-risk HEAR score patients in remote emergency medicine outposts in Bosnia and Herzegovina.

			Low-Risk HEAR Score (2 ≤ Points) N = 103	High-Risk HEAR Score (>2 Points) N = 338	Total N = 441
Sex (No, %)	Male		27 (26.3)	193 (57.2)	220 (49.9)
	Female		76 (73.7)	145 (42.8)	221 (50.1)
Age (mean ± SD)			50.5 ± 15.6	65.9 ± 12.1	63.5 ± 11.2
Risk factors (No, %)	Hypertemsion		40 (38.8)	307 (90.8)	347 (78.7)
	Diabetes mellitus		18 (17.4)	100 (29.6)	118 (26.8)
	Hyperlipidemia		38 (36.9)	265 (78.4)	303 (68.5)
	Stroke		1 (0.9)	5 (1.4)	6 (1.4)
	Peripheral vascular disease		2 (1.8)	25 (7.4)	27 (6.1)
	Smoking		22 (21.3)	123 (36.3)	145 (32.7)
		Never Smoker	81 (78.7)	215 (63.6)	296 (67.1)
		Quit	5 (4.8)	20 (5.9)	25 (5.7)
		Active Smoker	17 (16.5)	103 (30.4)	120 (27.2)
	Body mass index (BMI)				
		Underweight (<18.5 kg/m^2^)	1 (0.9)	0 (0.0)	1 (0.2)
		Normal (18.5–25 kg/m^2^)	75 (72.8)	240 (71.0)	315 (71.5)
		Overweight (25–30 kg/m^2^)	19 (18.4)	46 (23.6)	65 (14.8)
		Obese (>30 kg/m^2^)	8 (7.9)	52 (15.3)	60 (13.6)
	Family history		31 (30.0)	159 (47.0)	190 (43.1)

**Table 2:** Laboratory, blood pressure, and ECG findings among low-risk HEAR and high-risk HEAR score patients in remote emergency medicine outposts in Bosnia and Herzegovina.

		Low-Risk HEAR Score (2 ≤ Points) N = 103	High-Risk HEAR Score (>2 Points) N = 338
Laboratory findings (mean ± SD)	C-reactive protein	6.7 ± 3.2	8.0 ± 3.6
	D-dimer	0.5 ± 0.2	0.7 ± 0.3
	AST	45.0 ± 4.2	51.3 ± 4.7
	ALT	32.3 ± 8.3	35.7 ± 9.6
	CK	42.4 ± 12.3	53.2 ± 7.3
	CKMB	11.2 ± 4.3	15.2 ± 5.4
	LDH	253.4 ± 45.3	276.2 ± 49.2
	Urea	6.3 ± 4.2	9.3 ± 5.3
	Creatinine	88.3 ± 27.6	120.5. ± 45.2
Blood pressure (mean ± SD)	Systolic	136.1 ± 18.5	137.7 ± 21.3
	Diastolic	83.3 ± 8.4	82.9 ± 10.6
ECG findings (No, %)	Normal or nonspecific	80 (77.6)	78 (23.1)
	Nonspecific repolarization disturbance (LBBB or inverted T waves)	23 (22.4)	134 (39.6)
	Significant ST depression	0 (0)	126 (37.3)

AST—aspartate transaminase, ALT—alanine transaminase, CK—creatin kinase, CKMB—creatine kinase fraction MB, LDH—lactate dehydrogenase, LBBB—left bundle branch block.

**Table 3:** HEAR score and its components between low-risk HEAR and high-risk HEAR score patients.

			Low-Risk HEAR Score (2 ≤ Points) N = 103	High-Risk HEAR Score (>2 Points) N = 338	*p*-Value
HEAR SCORE	Patient history	Slightly suspicions	75 (72.8)	60 (17.7)	<0.001
		Moderately suspicions	26 (25.2)	183 (54.1)	
		Highly suspicious	2 (2.0)	95 (28.2)	
	ECG	Normal or nonspecific	80 (77.6)	78 (23.1)	<0.001
		Nonspecific repolarization disturbance (LBBB or inverted T waves)	23 (22.4)	134 (39.6)	
		Significant ST depression	0 (0)	126 (37.3)	
	Age	<45 years	64 (62.1)	57 (16.8)	<0.001
		45–65 years	36 (34.9)	156 (46.1)	
		>65 years	3 (3.0)	125 (37.1)	
	Risk factors	No risk facrtors known	80 (77.6)	34 (10.0)	<0.001
		1 or 2 risk factors	19 (18.4)	130 (38.4)	
		>3 risk factors/history of atherosclerosis disease	4 (4.0)	174 (51.6)	

## Data Availability

Data are available upon request.
